# Exogenous nitric oxide requires an endothelial glycocalyx to prevent postischemic coronary vascular leak in guinea pig hearts

**DOI:** 10.1186/cc6913

**Published:** 2008-06-02

**Authors:** Dirk Bruegger, Markus Rehm, Matthias Jacob, Daniel Chappell, Mechthild Stoeckelhuber, Ulrich Welsch, Peter Conzen, Bernhard F Becker

**Affiliations:** 1 Clinic of Anesthesiology, Ludwig-Maximilians-University, Marchioninistrasse 15, 81377 Munich, Germany; 2 Department of Anatomy, Ludwig-Maximilians-University, Pettenkoferstrasse 11, 80336 Munich, Germany; 3 Department of Physiology, Ludwig-Maximilians-University, Pettenkoferstrasse 12, 80336 Munich, Germany

## Abstract

**Introduction:**

Postischemic injury to the coronary vascular endothelium, in particular to the endothelial glycocalyx, may provoke fluid extravasation. Shedding of the glycocalyx is triggered by redox stress encountered during reperfusion and should be alleviated by the radical scavenger nitric oxide (NO). The objective of this study was to investigate the effect of exogenous administration of NO during reperfusion on both coronary endothelial glycocalyx and vascular integrity.

**Methods:**

Isolated guinea pig hearts were subjected to 15 minutes of warm global ischemia followed by 20 minutes of reperfusion in the absence (*Control *group) and presence (*NO *group) of 4 μM NO. In further experiments, the endothelial glycocalyx was enzymatically degraded by means of heparinase followed by reperfusion without (*HEP *group) and with NO (*HEP+NO *group).

**Results:**

Ischemia and reperfusion severely damaged the endothelial glycocalyx. Shedding of heparan sulfate and damage assessed by electron microscopy were less in the presence of NO. Compared with baseline, coronary fluid extravasation increased after ischemia in the *Control*, *HEP*, and *HEP+NO *groups but remained almost unchanged in the *NO *group. Tissue edema was significantly attenuated in this group. Coronary vascular resistance rose by 25% to 30% during reperfusion, but not when NO was applied, irrespective of the state of the glycocalyx. Acute postischemic myocardial release of lactate was comparable in the four groups, whereas release of adenine nucleotide catabolites was reduced 42% by NO. The coronary venous level of uric acid, a potent antioxidant and scavenger of peroxynitrite, paradoxically decreased during postischemic infusion of NO.

**Conclusion:**

The cardioprotective effect of NO in postischemic reperfusion includes prevention of coronary vascular leak and interstitial edema and a tendency to forestall both no-reflow and degradation of the endothelial glycocalyx.

## Introduction

Myocardial damage and coronary microvascular dysfunction, including the no-reflow phenomenon and edema formation, evolve from coronary occlusion and consecutive reperfusion. These are relatively common clinical occurrences, for example, in conjunction with percutaneous coronary angioplasty (PTCA), coronary artery bypass grafting (CABG), and heart transplant reperfusion [1]. Over the past 20 years, major advances have been made toward understanding the role of nitric oxide (NO) in the ischemic biology of the heart and it has become clear that NO, either endogenous or exogenous, represents one of the most important defenses against myocardial ischemia and reperfusion injury. The influence of NO on microvascular permeability is less clear because conflicting results exist in the literature [2,3].

A healthy vascular endothelium is coated by extracellular domains of a large variety of membrane-bound molecules that, together, constitute the glycocalyx. *In vivo*, the glycocalyx binds plasma proteins, forming the endothelial surface layer. This zone has a thickness of 400 to 500 nm in microvessels (in some regions, it is even thicker than the endothelial cells themselves) and is an integral part of the circulation. Various experimental models showed this large structure to be fundamentally involved in numerous physiological and pathophysiological actions in the circulation. Loss of coronary glycocalyx integrity is accompanied by leakage of fluid from the vascular compartment, resulting in swelling of the pericapillary interstitial space [4-6]. Moreover, it is recognized that the endothelial glycocalyx is especially prone to injury during ischemia/reperfusion [4,7,8]. Very recently, damage of the endothelial glycocalyx could be demonstrated in patients undergoing global or regional ischemia during vascular surgery [9]. Perturbation of the glycocalyx also results in adhesion of platelets and leukocytes to the capillary and venular endothelial surface [10,11]. Furthermore, coronary dilatation induced by shear stress also depends on an intact endothelial glycocalyx [12]. Based on these findings, there is little doubt that degradation of the endothelial glycocalyx leads to impaired regulation of organ blood flow, to activation of coagulatory and inflammatory pathways, and to tissue edema. Investigating strategies for protecting the endothelial glycocalyx against disruption obviously is an important topic for research.

Postischemic injury appears to depend substantially on the increased production of oxygen-derived free radicals upon reoxygenation. Redox stress may activate proteases, especially metalloproteases. Also, redox stress associated with ischemia/reperfusion induces mast cell degranulation, liberating tryptase and heparanase activity [13]. Activation and liberation of such enzymes will undoubtedly mediate disruption of the endothelial glycocalyx. Thus, by acting as a radical scavenger, NO administered in small amounts during reperfusion might help to protect the endothelium.

Given the established facts above, the present study investigated the effect of exogenous NO, given at the time of reperfusion, on the functional and metabolic outcome following myocardial ischemia/reperfusion, particularly with regard to the state of the endothelial glycocalyx. All experiments were conducted on an intact vascular bed, namely the coronary system of isolated perfused hearts (guinea pig Langendorff preparations), with a highly standardized protocol of ischemia and reperfusion. The impact of exogenous administration of NO (4 μM) was investigated by measuring coronary vascular resistance and net fluid filtration with and without enzymatic degradation of the glycocalyx by means of application of heparinase. The rationale for using heparinase is that it has proven to be specific for shedding heparan sulfates and that these structures comprise the greatest portion of the luminal endothelial glycocalyx [14]. The state of the glycocalyx was assessed by electron and light microscopy. The metabolic influence of NO was examined by measuring myocardial release of lactate, catabolism of adenine nucleotides, and degradation of the endogenous antioxidant uric acid during reperfusion.

## Materials and methods

This investigation conforms with the *Guide for the Care and Use of Laboratory Animals *[15]. Licensure and approval of the investigation were obtained from the Government of Upper Bavaria (file no. 209.1/211-2531.3-3/99).

### Heart preparation

In brief, guinea pigs (male; weight 250 to 300 g) were stunned by neck dislocation with a specially designed instrument, and, immediately after the opening of the thorax, the hearts were arrested with ice-cold isotonic saline. Quickly, the aorta was cannulated and the coronaries were perfused *in situ *at a constant flow rate of 6.0 mL/minute with a modified Krebs-Henseleit buffer (116 mM NaCl, 23 mM NaHCO_3_, 3.6 mM KCl, 1.16 mM KH_2_PO_4_, 1.2 mM CaCl_2_, 0.58 mM MgSO_4_, 5.4 mM glucose, 0.3 mM pyruvate, and 2.8 U/L insulin) oxygenated with 94.5% O_2 _and 5.5% CO_2 _at 37°C, pH 7.40 ± 0.05. Hearts were removed from the thorax and prepared especially for quantifying net coronary fluid leak as described previously [5,6,16,17]. The hearts were not inverted and beat spontaneously. Such hearts are not readily suited for determining contractile function. Coronary perfusion pressure in the aortic feed line was registered online (pressure transducer; Seeheim-Ober Beerbach, Engelsbach, Germany). Coronary venous effluent was collected from the pulmonary artery, cannulated and ending with a slightly negative hydrostatic pressure to preclude coronary venous congestion. Interstitial and lymphatic fluids formed by net filtration appeared at the epicardial surface and dripped off the apex of the heart. This so-called transudate was collected at 1-minute intervals and quantified by weighing on a precision scale. This value was subsequently divided by the ventricular wet weight (w.w.) so that the unit of transudate formation is expressed as milliliter per minute per gram of w.w. Samples of effluent and transudate were then immediately frozen.

Infusion lines were inserted into the aortic feed line just above the coronary ostia to infuse small aliquots of a saturated aqueous solution of NO (see below) or of heparinase type-1 enzyme (Sigma-Aldrich, St. Louis, MO, USA). We have recently shown by electron microscopy, immunohistological staining, and quantitative measurement of constituent parts of the glycocalyx that this enzyme selectively sheds heparan sulfate from the endothelial glycocalyx [14]. Enzymograms (PAGE, gelatine) showed that heparinase had no collagenase activity versus control, excluding enzyme activity, for example, against the basement membrane. Constant-flow instead of constant-pressure perfusion was chosen for the current experimental model to guarantee constant levels of infused substances. The protocol allowed infusion of NO stock solution calculated to yield an arterial infusate concentration of 4 μM.

### Control experiments

The heart preparations were first characterized without ischemia. Time control experiments were performed to evaluate the influence of 15-minute equilibration and 20-minute perfusion without ischemia on coronary perfusion pressure and on transudate formation (*Time*_*Control *_group). Moreover, time-matched control values were measured after 20-minute administration of 4 μM NO (*NO*_*Control *_group) and after 15-minute application of 10 U of heparinase (*Hep*_*Control *_group). The results are presented in Table 1. NO infused in this manner did not elicit vasodilatation; the threshold value required to do so was approximately 10 μM (nominal arterial infusate concentration). The application of heparinase caused a measurable increase in transudate formation.

**Table 1:** Control experiments without ischemia

	*Time*_*Control *_(n = 5)	*NO*_*Control *_(n = 5)	*HEP*_*Control *_(n = 6)
Coronary perfusion pressure, percentage	114 ± 7	89 ± 4	119 ± 5
Transudate formation, percentage	128 ± 17	99 ± 14	260 ± 14^a^

Values are presented as a percentage of basal and as mean ± standard deviation. The groups are as defined in Materials and methods. ^a^*P *< 0.05, intragroup difference versus basal.

### Experimental protocols

**Figure 1:**
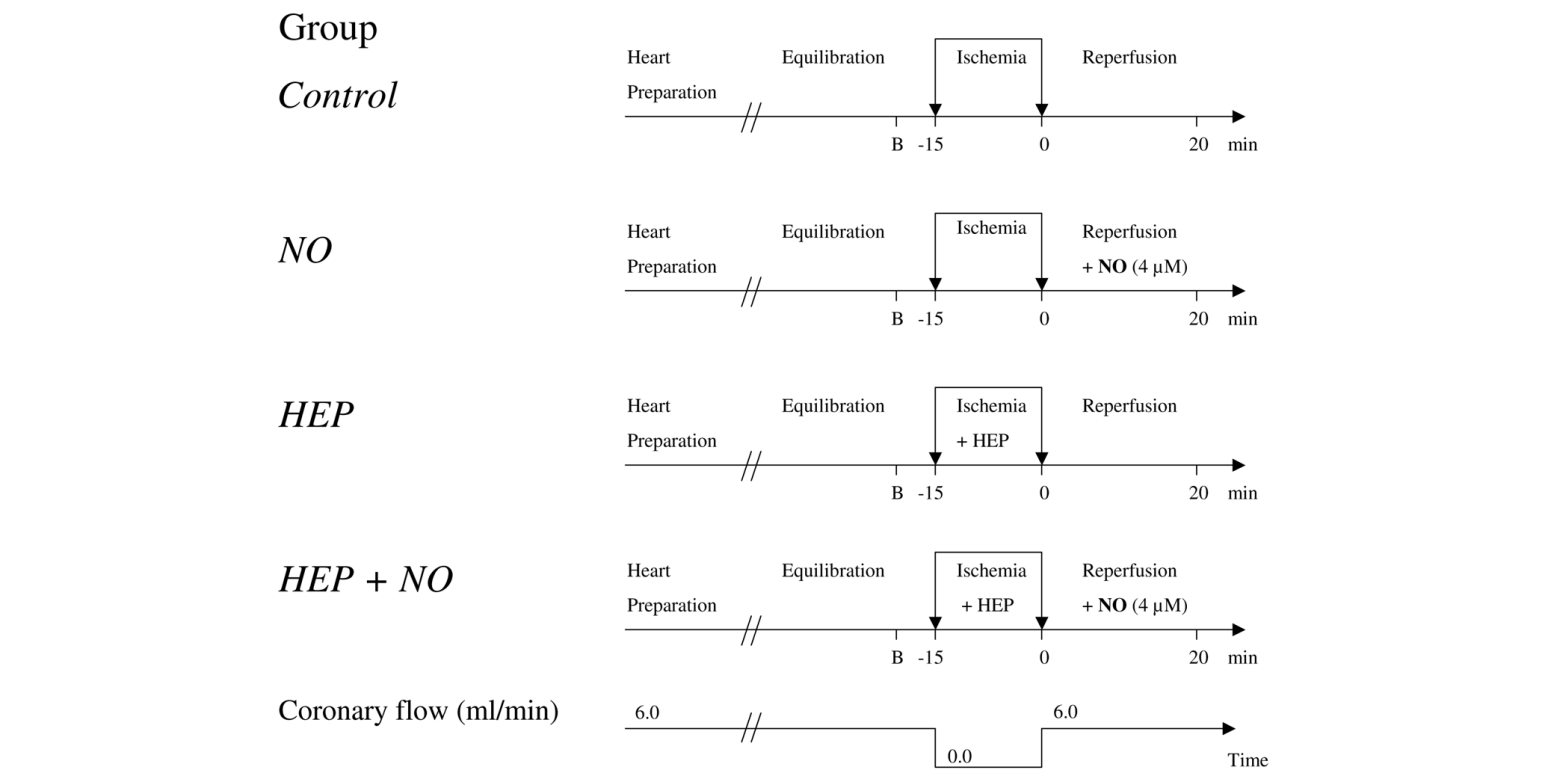
Experimental protocols. After heart preparation and an equilibration period of 15 minutes, hearts were subjected to 15 minutes of global normothermic ischemia followed by 20 minutes of reperfusion. Hearts were reperfused in the absence (*Control *group) and presence (*NO *group) of 4 μM nitric oxide. In two further series, the glycocalyx was enzymatically degraded by heparinase, applied into the coronary system in the course of ischemia, followed again by reperfusion in the absence (*HEP *group) and presence (*HEP+NO *group) of 4 μM NO. Transudate and coronary effluent were quantified at baseline (B) (just before ischemia) and at 1, 2, 3, 4, 5, 8, 15, and 20 minutes of reperfusion. HEP, heparinase.

Hearts were randomly assigned among the experimental groups. Figure 1 illustrates the protocols applied to four groups of hearts. The standard condition was that of constant coronary flow perfusion (6.0 mL/minute) at 37°C. After an equilibration period of 15 minutes, hearts were subjected to 15 minutes of global, normothermic, stopped-flow ischemia and then reperfused under the same condition as before ischemia. To maintain the temperature of the preparation during ischemia, hearts were immersed in warm Tyrode's buffer (37°C). Hearts were reperfused in the absence of NO (Figure 1, *Control *group), in the presence of 4 μM NO (*NO *group), and after the glycocalyx had been enzymatically degraded by means of heparinase (10 U of enzyme in a volume of 1.5 mL applied into the coronaries in the course of the 15-minute ischemic phase), both without NO (*HEP *group) or with 4 μM NO (*HEP+NO *group). Samples of transudate and coronary effluent were taken at baseline and during reperfusion. At the end of each experiment, hearts were removed from the perfusion system and the atria and large vessels cut away. Excess surface and intraventricular fluid was swabbed off and the ventricles weighed at once.

### Preparing and applying NO stock solution

To prepare an aqueous saturated solution of NO, 3 mL of deionized water was sealed into a small glass vial with a stopper and a long syringe needle was inserted, extending almost to the base of the vial. An additional shorter needle that did not immerse into the solution was inserted and acted as a pressure relief. After the system was purged with argon (Linde AG, Munich, Germany) for a period of 30 minutes at a moderate flow rate, the system was bubbled for 30 minutes with pure NO (Linde AG). Subsequently, the needles were removed and the rubber stopper sealed spontaneously. Just before the experiment, this solution was transferred under exclusion of oxygen into a glass syringe equipped with a long metal cannula. The concentration of NO resulting from saturation at 20°C is approximately 1.91 mM.

### Quantification of tissue edema

At the end of each protocol, tissue edema was assessed by measuring ventricular wet weight (at once) and dry weight (after 24 hours of drying at 60°C). The quotient 'wet weight/dry weight' was then calculated.

### Determination of release of lactate, purines, and uric acid

Coronary venous effluent of the *Control *and *NO *groups was collected via the pulmonary artery, and aliquots were stored (-20°C) until analyzed. The concentration of lactate was determined by high-performance liquid chromatography (HPLC) in aliquots acidified (pH 2.0) with perchloric acid [18]. The total concentrations of the purines adenosine, inosine, hypoxanthine, xanthine, guanosine, and urate together (products of adenine nucleotide catabolism) as well as that of uric acid alone were quantified by reverse-phase HPLC [19]. In each case, the concentration value, multiplied by the coronary flow rate and divided by the ventricular w.w., yielded the respective rate of cardiac release per gram of heart weight. The cumulative release of purines was established over the first 15 minutes of reperfusion by calculating an area under the curve using the trapezoidal method (Sigma plot; SPSS Inc., Chicago, IL, USA).

### Determination of heparan sulfate

After 15 minutes of ischemia, samples of effluent were collected at 5, 8, 15, and 20 minutes during reperfusion and were used for assessing shedding of heparan sulfate. To aggravate the ischemia/reperfusion injury, additional hearts were subjected to 20 minutes of ischemia followed by 40 minutes of reperfusion. All samples were preconcentrated over 10-kDa cutoff membrane filters (Millipore, Eschborn, Germany). Concentrations were determined with an enzyme-linked immunosorbent assay (Seikagaku Corporation, Tokyo, Japan). The cumulative release was established by calculating an area under the curve (see above).

### Light microscopy and immunohistochemistry

Hearts were perfusion-fixed after minimal perfusion (<1 minute), after 15 minutes of ischemia followed by 20 minutes of reperfusion in the absence and presence of NO, and after the glycocalyx had been enzymatically degraded by means of heparinase in the course of the ischemic phase. Fixation occurred by the addition of formaldehyde to the flowing Krebs-Henseleit buffer to a resulting concentration of 1%. After 4 minutes, the hearts were removed from the apparatus and stored in 4% formaldehyde solution for 24 hours. Paraffin sections (5 μm) were immunohistochemically stained with monoclonal antibody against heparan sulfate (Seikagaku Corporation). The primary antibody, applied to generate an avidin-biotin horseradish peroxidase complex with the Vectastain kit (Vector Laboratories, Burlingame, CA, USA), was diluted and handled as follows: anti-heparan sulfate 1:100, tissue preincubation with 0.2% trypsin at 37°C. Controls in which the primary antibody was replaced with buffer were treated identically. Diaminobenzidine or aminoethylcarbazole was used as chromogen.

### Electron microscopy

Electron microscopy, performed in modification of Vogel and colleagues [20], was based on *in situ *stabilization of the glycocalyx by intracoronary application of a fixative containing lanthanum ions and glutaraldehyde [5,6,16,17].

### Statistical analysis

Because the measured data were distributed normally (determined by Kolmogorov-Smirnov test), these are presented as mean ± standard deviation unless otherwise indicated. Comparisons were made using analysis of variance for repeated measurements. *Post hoc *testing was performed using the Student-Newman-Keuls method for multiple comparisons. A *P *value of less than 0.05 was considered to be significant.

## Results

**Figure 2:**
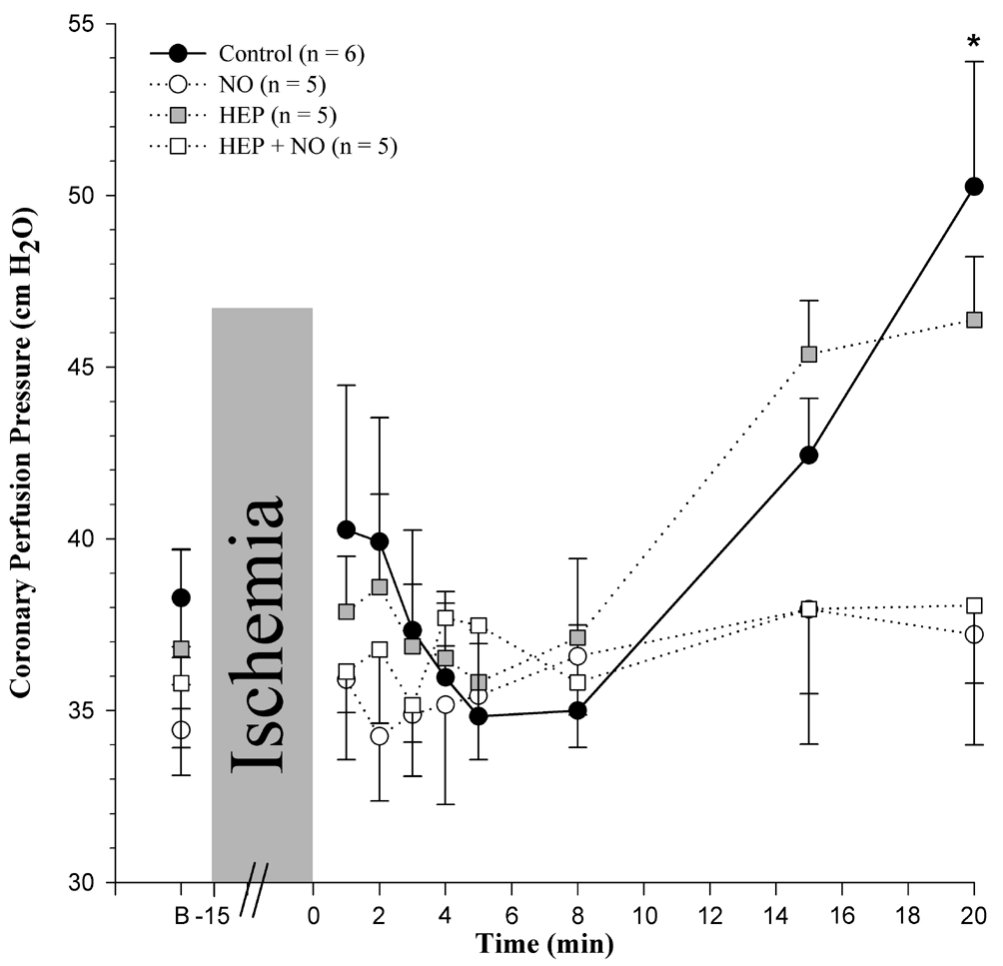
Effect of ischemia/reperfusion on coronary perfusion pressure. Groups are as defined in the legend of Figure 1. Values are presented as mean ± standard error of the mean. *P < 0.05, intragroup difference versus basal (B).

The effect of ischemia and reperfusion on coronary perfusion pressure is shown in Figure 2 for all experimental groups. In the *Control *and *HEP *groups, the data reveal that a transient postischemic vasodilatation (decrease in coronary perfusion pressure) tended to develop approximately 5 minutes after the end of ischemia. This was followed by vasoconstriction (increase in coronary perfusion pressure). After 20 minutes of reperfusion, coronary perfusion pressure had increased significantly versus baseline in the *Control *group. In the presence of 4 μM NO (*NO *and *HEP+NO *groups), this latter increase in coronary perfusion pressure did not occur. Although there were no significant intergroup differences after 20 minutes, there was a significantly lower coronary resistance for all hearts with NO as compared with those without (*P *< 0.05, pooled data, n = 10 and 11, respectively). Pertinently, coronary resistance did not increase within the first 5 minutes when the infusion of NO was terminated after 20 minutes (results not shown). Thus, there was no acute and direct dilatatory effect of NO.

**Figure 3:**
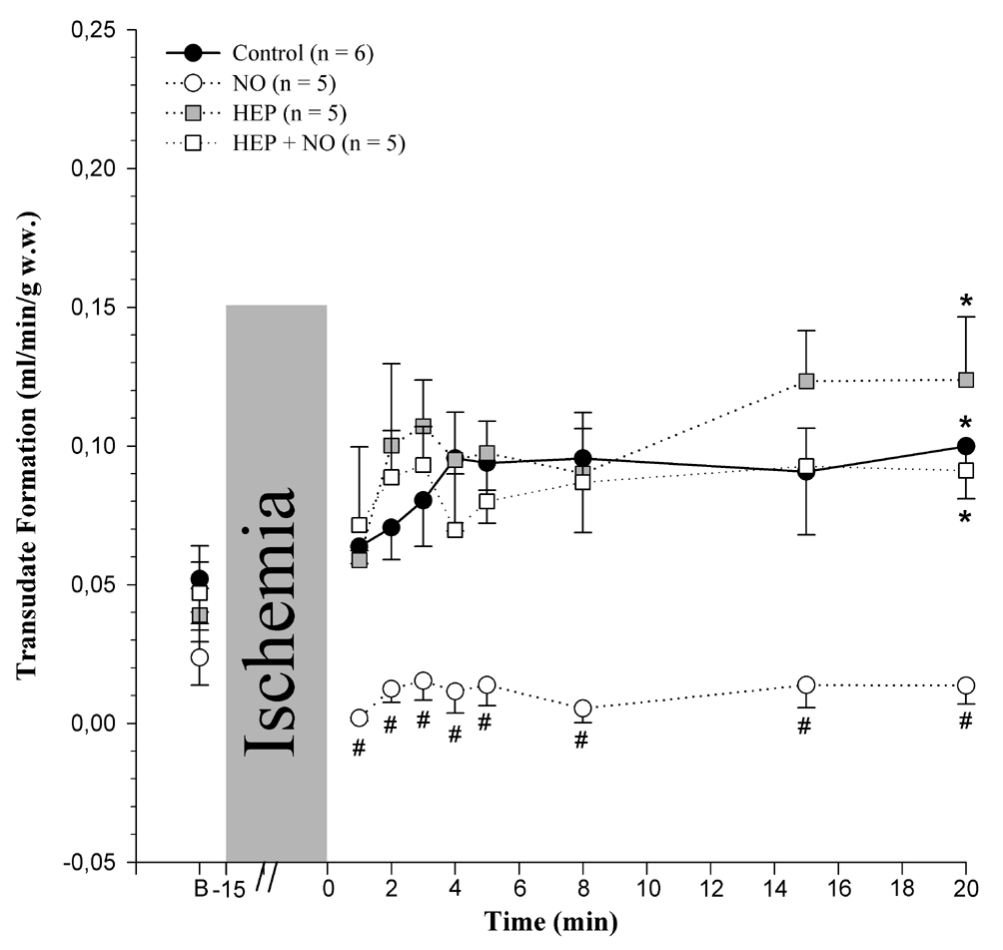
Effect of ischemia/reperfusion on coronary transudate formation. The groups are as defined in the legend of Figure 1. Values are presented as mean ± standard error of the mean. *P < 0.05, intragroup difference versus basal (B); ^#^*P *< 0.05, intergroup difference versus the *Control *group. w.w., wet weight.

Postischemic changes in transudate formation (that is, of net fluid filtration in the intact coronary bed of isolated guinea pig hearts) are illustrated in Figure 3 for all experimental groups. Baseline transudate formation – about 20 to 50 μL/minute per gram of w.w. – did not differ significantly among the four groups. Postischemically, there was an increase in transudate formation in the *Control*, *HEP*, and *HEP+NO *groups with respect to the individual group baseline (Δ = +50, +85, and +44 μL/minute per gram of w.w. after 20 minutes, respectively). In contrast, when shedding of heparan sulfate had not been enzymatically induced prior to reperfusion (see below), transudate formation remained almost unchanged when 4 μM NO was applied during reperfusion (Δ = -13 μL/minute per gram of w.w. after 20 minutes) and was significantly lower than in the *Control *group at all times of reperfusion (Figure 3). Interestingly, no correlation was found between transudate formation and coronary perfusion pressure (R^2 ^= 0.016; n = 189, pooled values of 21 hearts at 9 measuring time points each), also demonstrating that changes in transudate formation were not the result of any putative acute vasodilatatory effect of NO.

Mean wet-to-dry weight ratios of the isolated heart preparations determined at the end of each experimental protocol are listed in Table 2. The mean ratios for hearts of *Control*, *HEP*, and *HEP+NO *groups ranged from 7.9 to 8.3. NO applied to hearts without foregoing treatment with heparinase (*NO *group) yielded a mean ratio of 6.9, indicating a significant decrease in tissue edema formation.

**Table 2:** Quantification of tissue edema

Group	Wet weight/dry weight
Control experiments	
*Time*_*Control *_(n = 5)	7.0 ± 0.7
*NO*_*Control *_(n = 5)	7.0 ± 0.6
*HEP*_*Control *_(n = 6)	8.5 ± 0.6
Experimental groups	
*Control *(n = 6)	8.0 ± 0.3
*NO *(n = 5)	6.9 ± 0.2^a^
*HEP *(n = 5)	8.3 ± 0.6
*HEP+NO *(n = 5)	7.9 ± 0.6

Values are presented as mean ± standard deviation. ^a^*P *< 0.05 with respect to *Control*, *HEP*, and *HEP+NO *groups.

**Figure 4:**
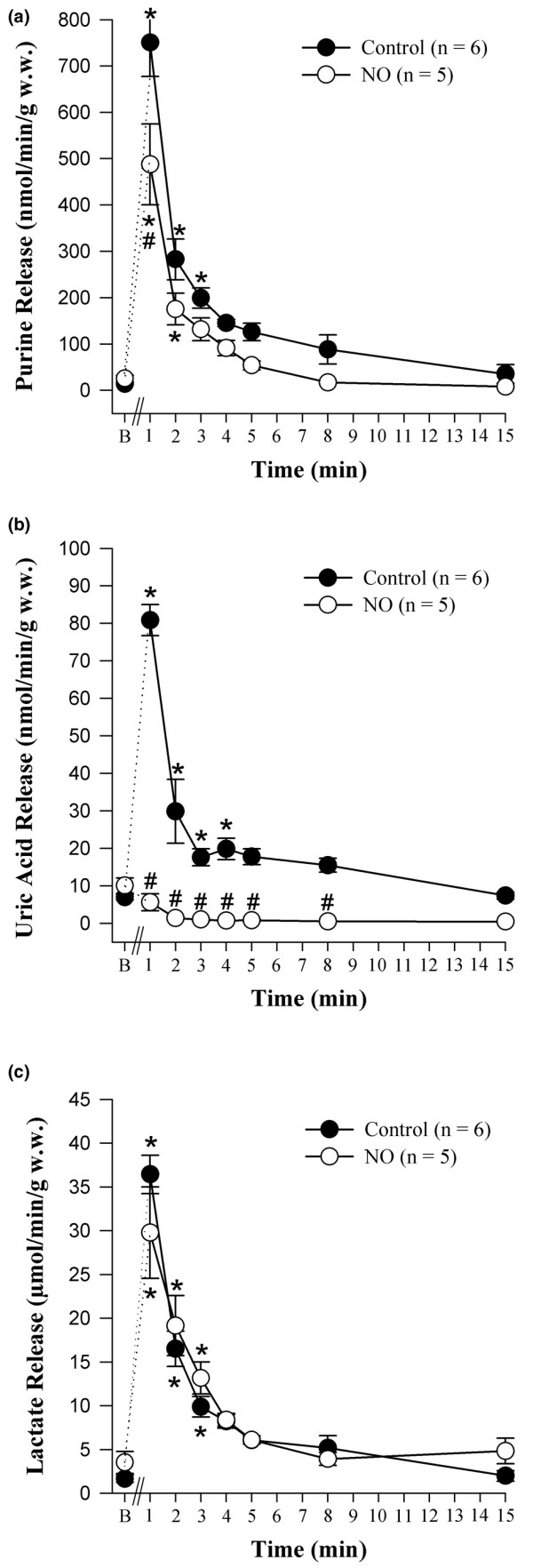
Coronary venous release of metabolites during the first 15 minutes of myocardial reperfusion. **(a) **Release of purines. **(b) **Release of urate. **(c) **Release of lactate. Hearts were reperfused in the absence (*Control *group) and presence (*NO *group) of 4 μM nitric oxide. Values are presented as mean ± standard error of the mean. *P < 0.05, intragroup difference versus basal (B); ^#^*P *< 0.05, intergroup difference versus the *Control *group. w.w., wet weight.

Metabolic data were determined in the absence (*Control *group) and presence (*NO *group) of 4 μM NO. The release of purine compounds in the coronary effluent serves as a quantitative measure of adenine nucleotide catabolism in the heart and is illustrated in Figure 4a. A beneficial effect of NO was observed: acute postischemic release of purines was reduced in its presence and cumulative purine release during reperfusion was almost halved (*Control *group: area under the curve = 1,907 ± 517 nmol per gram of w.w.; *NO *group: area under the curve = 1,097 ± 427 nmol per gram of w.w.; *P *< 0.05, n = 6 and 5, respectively). This effect exceeded by far a decrease in the net amount of urate released from these hearts: postischemic release of uric acid (Figure 4b) paralleled that of total purines in the *Control *group, increasing more than eightfold during early reperfusion. However, it accounted for only about 10% of the total release of purines in the *Control *group. In the presence of NO, urate decreased significantly to only a few percent of the total purine release throughout. Lactate release (Figure 4c) increased more than 10-fold during early reperfusion in both the *Control *and *NO *groups and was not significantly changed by the presence of NO during the subsequent reperfusion. There was no difference in the cumulative release of lactate (area under the curve) between both groups.

**Figure 5:**
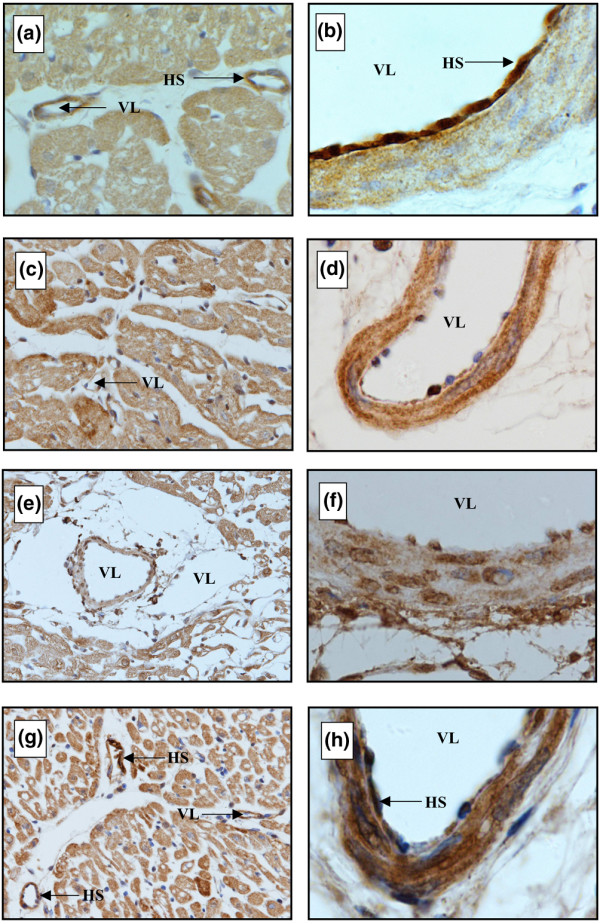
Immunostaining of microvessels of guinea pig hearts with monoclonal antibody against heparan sulfate after minimal perfusion **(a, b)**, after 15 minutes of ischemia followed by 20 minutes of reperfusion in the absence **(c, d) **and presence **(g, h) **of nitric oxide, and after the glycocalyx had been enzymatically degraded by means of heparinase **(e, f)**. Specimens are paraffin-embedded sections of guinea pig heart. Original magnifications: × 20 (lefthand panels) and × 100 (righthand panels). HS, heparan sulfate; VL, vascular lumen.

Light microscopy after immunohistochemical staining of nonischemic hearts evidenced heparan sulfate as a component of the endothelial glycocalyx (Figures 5a and 5b, respectively). In hearts subjected to ischemia and reperfusion without NO, the staining of the endothelial cell lining was markedly reduced (Figures 5c and 5d, respectively). In hearts that were reperfused after the glycocalyx had been enzymatically degraded by means of heparinase, no heparan sulfate was detected at the endothelial cell lining (Figures 5e and 5f, respectively). Hearts reperfused in the presence of NO showed some positive staining for heparan sulfate after 20 minutes of reperfusion (Figures 5g and 5h, respectively). Interestingly, the walls of the vasculature also contained heparan sulfate.

**Figure 6:**
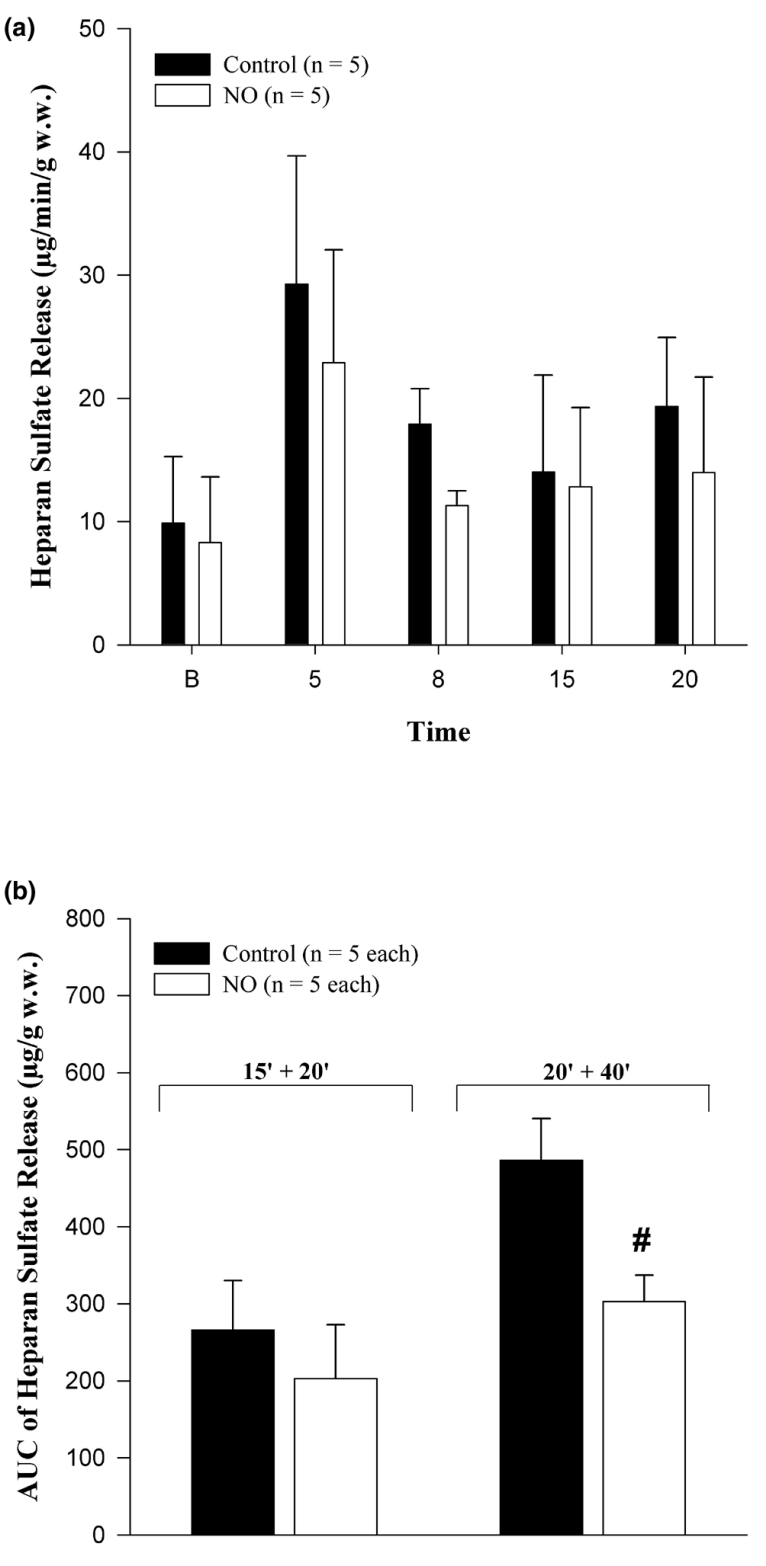
Heparan sulfate release in coronary venous effluent. **(a) **Absolute release of heparan sulfate in hearts subjected to 15 minutes of ischemia followed by 20 minutes of reperfusion. **(b) **Cumulative release of heparan sulfate in hearts subjected to 15 minutes of ischemia followed by 20 minutes of reperfusion (15' + 20') and in hearts subjected to 20 minutes of ischemia followed by 40 minutes of reperfusion (20' + 40'). Hearts were reperfused in the absence (*Control *group) and presence (*NO *group) of 4 μM nitric oxide. Values are presented as mean ± standard error of the mean. ^#^*P *< 0.05, intergroup difference versus the *Control *group. AUC, area under the curve; B, baseline; w.w., wet weight.

Material immunopositive for heparan sulfate was detected in the coronary effluent of the isolated perfused hearts and a postischemic increase was observed after 15 minutes of ischemia (Figure 6a). There was a tendency toward a lower cumulative release of heparan sulfate during reperfusion in the presence of NO but this lacked statistical significance. However, extending the ischemic period to 20 minutes followed by 40 minutes of reperfusion showed a significantly lower cumulative release of heparan sulfate during reperfusion in the presence of NO.

**Figure 7:**
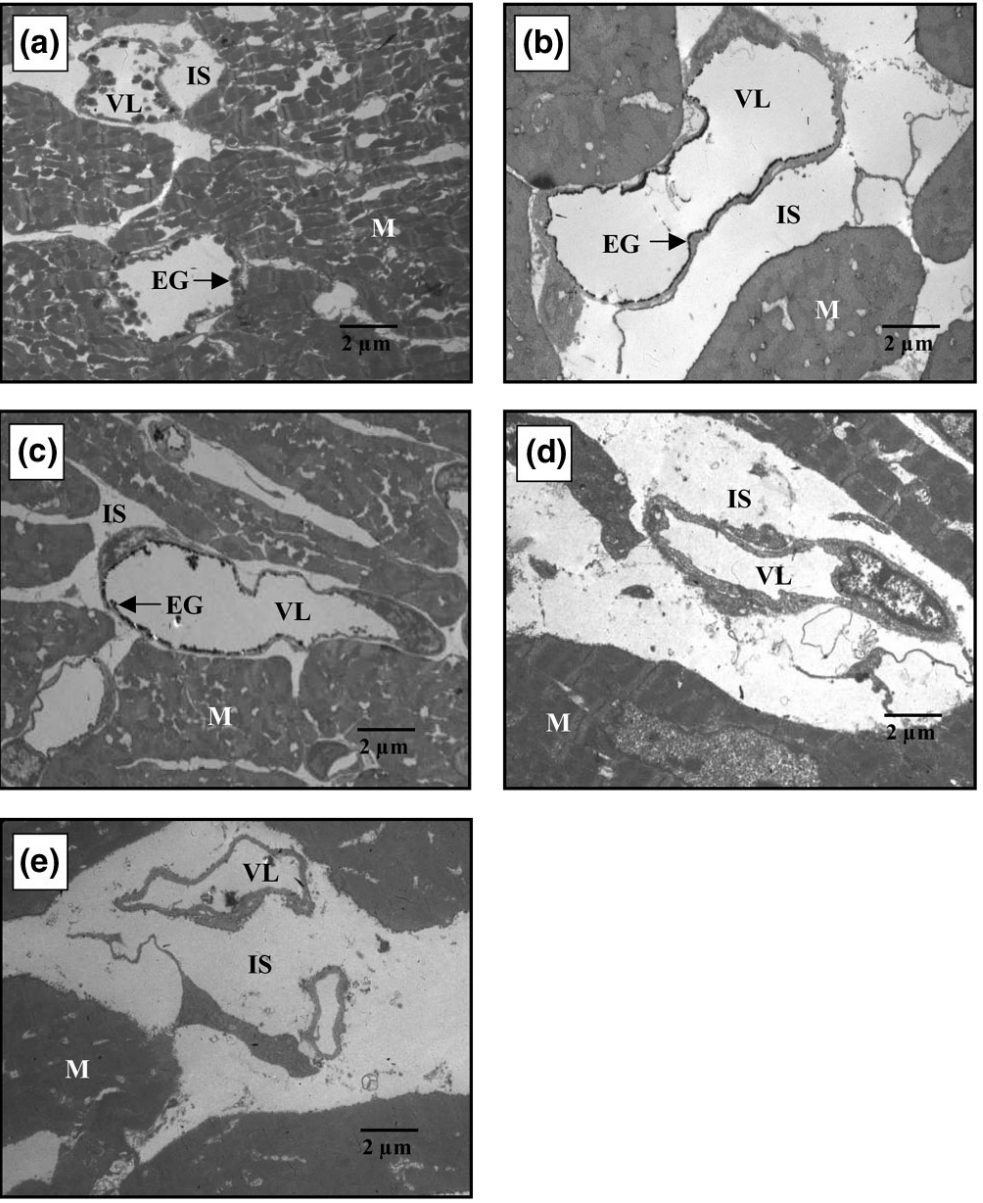
Exemplary electron microscopic images of hearts without ischemia **(a) **and hearts subjected to 15 minutes of warm ischemia and 20 minutes of reperfusion without nitric oxide (NO) **(b) **(*Control *group) and with 4 μM exogenous NO **(c) **(*NO *group). The glycocalyx of 200 to 300 nm in width normally present in all vessels has virtually disappeared after ischemia and reperfusion in the absence of NO and edema has developed. NO partly mitigated these effects. The effect of heparinase is shown in **(d) **(*HEP *group) and **(e) **(*HEP+NO *group). EG, endothelial glycocalyx; IS, interstitial space; M, cardiomyocytes; VL, vascular lumen.

Exemplary electron microscopic images illustrating coronary vessels of the isolated guinea pig hearts are shown in Figure 7. In comparison with a nonischemic heart, where an endothelial glycocalyx of approximately 200- to 300-nm thickness was found in the coronary vasculature, hearts subjected to ischemia and reperfusion showed a dramatic loss of the endothelial surface lining and strong edema formation (Figures 7a and 7b, respectively). Hearts that had received NO during reperfusion exhibited less edema, but the glycocalyx still showed partial denudation (Figure 7c). Hearts pretreated with heparinase during ischemia were completely denuded, irrespective of the presence of NO during reperfusion (Figures 7d and 7e, respectively).

## Discussion

The major findings of this study are that NO, applied with the onset of reoxygenation, protects isolated hearts against some facets of postischemic reperfusion injury and that this protective effect seems to extend to the endothelial glycocalyx in the coronary vascular bed. The evidence provided by this investigation is the following:

1. Coronary resistance tended to increase during reperfusion, but not if NO was applied. This effect seemed to be maintained even if the glycocalyx had been degraded beforehand.

2. Fluid extravasation in the coronary system was increased after ischemia, but not if NO was applied during reperfusion. The postischemic increase in tissue edema was alleviated if hearts were reperfused in the presence of NO. NO had no preventive effect on coronary leak or edema if the endothelial glycocalyx was degraded beforehand.

3. NO attenuated shedding of the endothelial glycocalyx, as judged by enzyme-linked immunosorbent assay and electron microscopy.

4. Metabolic stress was mitigated during reperfusion with NO, as evidenced by a significantly lowered catabolism of high-energy adenine nucleotides.

The observed trends in change of coronary perfusion pressure comply with the no-reflow phenomenon and may be explained in part by the changing absolute availability of endogenous NO during reperfusion. The concentration of endogenous NO in the coronary effluent, about 0.2 μM during normoperfusion [21,22], increases again from zero during early reperfusion [23]. However, with prolongation of reperfusion, NO synthase activity decreases and NO is also consumed by reaction with superoxide and hydroxyl radicals. Thus, NO decreases below baseline values [22]. These expected changes in NO concentration parallel the changes in coronary perfusion pressure observed in the present study: hearts reperfused in the absence of NO (*Control *and *HEP *groups) showed an intermittent decrease in coronary perfusion pressure followed by an increase. Such an increase did not occur in hearts reperfused with exogenous NO (*NO *and *HEP+NO *groups), although NO applied into the arterial perfusate at this level (4 μM) did not *per se *elicit coronary dilatation. Since coronary perfusion pressure directly reflects coronary resistance under conditions of constant coronary flow as used here, it seems that moderate amounts of exogenous NO (see below) may be able to compensate the increase in vascular tone caused by depletion of endogenous NO in reperfused hearts. However, alternative actions, not directed immediately toward the vascular smooth muscle, also need to be considered. One such possibility would be alleviation of interstitial edema (that is, of extramural compression). This was found only for the *NO *and not for the *HEP+NO *hearts, suggesting that interstitial pressure was not a major determinant of coronary resistance. Despite the seemingly good concordance of effect and expectation concerning NO levels, the actual magnitudes of the coronary resistance changes seen in the present study were definitely limited. Thus, the action of NO in this respect should not be overstated.

A far stronger action was evident concerning vascular leak. In the isolated perfused heart model, changes in transmural fluid passage in the entire coronary bed can be assessed directly by determining the flow of transudate. This fluid, which appears on the epicardial surface, may be regarded as a mixture of interstitial and lymphatic liquid and its flow is equal to net fluid filtration in the coronary system. The basal value of up to 50 μL/minute per gram of w.w. obtained in the present study compared favorably with that reported by others [24,25], as did the observed postischemic increase. The endothelial glycocalyx plays a major role in the development of postischemic coronary vascular leak [8,26] and, together with the endothelial cells themselves, constitutes the vascular 'double barrier' against fluid extravasation [16,17].

Bulk permeability of water and small hydrophilic solutes is basically influenced by the Starling forces of intravasal and extravasal hydrostatic and oncotic pressures and by the vascular barrier. The only variable force in our model was the coronary perfusion pressure. However, there was certainly no consistent relationship between coronary perfusion pressure and transudate formation in the four groups of hearts (compare Figures 2 and 3). Thus, the observed differences of net fluid transport across the vascular wall at any one time must reflect differences in hydraulic conductivity (that is, in the integrity of the double barrier composed of the endothelial glycocalyx and the endothelial cells). When the glycocalyx is damaged, vascular permeability increases. This was seen in hearts treated with heparinase (Table 1: *HEP*_*Control*_; Figure 2: *HEP *and *HEP+NO *groups). The elevated formation of transudate after 15 minutes of ischemia in hearts not treated with heparinase (*Control *group) supports the view that ischemia/reperfusion damaged the glycocalyx. In agreement with the assumption that NO alleviated degradation of the coronary endothelial glycocalyx, there was less leak and less heparan sulfate-reactive material tended to appear in the coronary effluent of those hearts reperfused solely with 4 μM NO. Electron microscopic visualization of the microvasculature in control hearts and hearts subjected to ischemia/reperfusion without and with NO corroborated the partial protection of the endothelial glycocalyx by exogenous NO (Figure 7).

There has been an ongoing debate in the literature as to whether NO is a protectant or a source of injury in the reperfused heart, but it has clearly been demonstrated that NO has infarct-sparing abilities in isolated buffer-perfused hearts [27]. In line with the results of the present study, various drugs (for example, statins, angiotensin-converting enzyme inhibitors, angiotensin-receptor blockers, nitrates, and nitrites) have produced beneficial effects in experimental models of ischemia/reperfusion via the enhancement of NO bioavailability [28]. Cardioprotection by inhalational NO has not been conclusively demonstrated in humans. However, the NO donor nitroprusside improved coronary flow and myocardial pump function in patients undergoing elective PTCA [29] and CABG [30,31], respectively.

The metabolic data also indicate further protective actions of NO in the isolated guinea pig hearts. The release of purines has been shown to be a sensitive and accurate index of energy metabolism [32]: purine release increases whenever energy production is impaired or inadequate to meet demand and decreases if energy balance is restored. It may be postulated that NO mitigates energy demand resulting from oxidative stress (for example, the familiar postischemic dysregulation of cytosolic ion levels). Irrespectively, the observed preservation of purines in hearts reperfused with NO will serve to restore and/or maintain levels of adenine nucleotides and high-energy phosphates because *de novo *synthesis of purines – the only alternative to salvage – is an energetically very costly process.

In contrast, washout of lactate did not differ between the *Control *and *NO *groups. Thus, direct ischemic stress was comparable in the two groups and, as was to be expected, is uninfluenced by NO applied afterwards. This result is in full accord with the study postulate.

Microvascular permeability induced by ischemia/reperfusion in the dog heart was reduced by antioxidants and radical scavengers [33]. A protective effect of NO could reside in an enhanced scavenging of reactive oxygen species, particularly hydroxyl and superoxide radicals. Indirect evidence for such an effect of NO can be derived from the substantially lower concentration of uric acid found in the effluent of hearts reperfused with NO. Uric acid reacts avidly with peroxynitrite formed by reaction of NO with superoxide [18]. The enhanced formation of urate due to nucleotide catabolism in hearts subjected to ischemia/reperfusion (see *Control *group) actually provides more endogenous antioxidant to mitigate the removal of peroxynitrite. It should be kept in mind that humans have exceptionally high blood urate levels [34]. The generation of physiological antioxidants such as urate in complete biological systems allows NO to alleviate postischemic redox stress without giving rise to toxic products [19,34]. Although the data certainly show a change in the stress to hearts of the *NO *group as opposed to the *Control *group (compatible with a scavenging of radicals and reactive oxidative species), the metabolic effects seen in these hearts could also arise from G-protein- and c-GMP-induced actions or from other cellular effects of NO [27,28].

A critique of the experimental model used here should address the likely partial oxidation of NO applied into an oxygenated Krebs-Henseleit perfusate. Although the time of contact from the site of infusion to entry into the coronary system is only about 0.2 seconds, some of the NO will react with oxygen to form toxic nitrogen dioxide (NO_2_) and higher oxides. A similar fate befalls NO applied via the respiratory tract in human therapy. Like peroxynitrite, NO_2 _reacts with urate. Thus, part of the marked decrease in coronary urate levels seen in our perfusion protocol upon application of NO may well reflect oxidative degradation due to inactivation of NO_2_. Owing to the high plasma levels of urate in humans, the same detoxification may be expected when NO is used clinically, explaining its relative safety in human use [35].

We had deliberated extensively on alternative routes and sources of NO before deciding on the application of physically dissolved, pure NO. A major drawback of chemical generators of NO, such as NO-penicillamine, S-nitrosoglutathione, and SIN-1, is that only the rate of generation of NO can be predicted, but not the actual concentration in the solution being acutely applied, as hydrolysis to NO and oxidation of NO are occurring from the moment water is added to compose the respective stock solutions. On the other hand, the concentration of NO in a saturated aqueous solution at room temperature and normal atmospheric pressure is well characterized in the literature. Moreover, the choice of direct NO generators in clinical use is neither very great nor tempting.

Action on the combined barrier formed by the glycocalyx and the endothelial cell bodies is better assessed in the presence of colloidal substances [6]. Colloids are able to establish an oncotic gradient at the endothelial surface, opposing fluid filtration [17]. However, postischemic application of hydroxyethyl starch alone to the perfusate already prevented much of the increase in coronary leak that developed during reperfusion [36].

Because the isolated hearts are perfused with blood-free Krebs-Henseleit buffer solution, the endothelial cells are presumably covered only by membrane-anchored molecules. That is, the glycocalyx in its strictest sense, and not the endothelial surface layer, is under investigation. It is also impossible to assess aspects of ischemia/reperfusion injuries that may be initiated and influenced by plasma-borne factors and formed constituents of blood. On the other hand, physiologic antioxidants of plasma are absent, perhaps accentuating damage by reactive oxygen species. Inhalational NO has been shown to be beneficial after myocardial infarction [37], but no link was drawn to the glycocalyx. This is quite understandable because it is not easy to directly visualize the glycocalyx *in vivo*. Thus, *in vitro *models are still required.

## Conclusion

In summary, the present study shows a protective role for NO during myocardial reperfusion. The cardioprotective effect of NO in ischemia/reperfusion includes attenuation of the coronary no-reflow phenomenon and prevention of coronary vascular leak and tissue edema. The latter actions are based on preservation of the endothelial glycocalyx.

## Key messages

• Nitric oxide attenuates the increase in coronary leak and edema formation induced by ischemia/reperfusion.

• This action is based on protection of the endothelial glycocalyx against shedding.

## Abbreviations

CABG = coronary artery bypass grafting; HPLC = high-performance liquid chromatography; NO = nitric oxide; NO_2 _= nitrogen dioxide; PTCA = percutaneous coronary angioplasty; w.w. = wet weight.

## Competing interests

The authors declare that they have no competing interests.

## Authors' contributions

DB was responsible for acquisition of data, analysis and interpretation of data, and drafting the manuscript. MR conceived of the study and participated in data interpretation and manuscript development. DB and MR contributed equally. MJ and DC took part in all animal experiments performed and were responsible for surgical preparation and data collection. MS and UW performed the electron microscopy, light microscopy, and immunohistochemistry. PC analysed and interpreted the data. BFB was responsible for the study design and the analysis and interpretation of data and helped to draft the manuscript. All authors have read and approved the final manuscript.
